# Arrhythmias as Part of Long COVID Syndrome in Hospitalized Patients That Survived a Severe COVID-19 Infection and the Potential Protective Role of Metformin in These Patients

**DOI:** 10.3390/life16020319

**Published:** 2026-02-12

**Authors:** Haydee Ninette Morales-Vazquez, David Cardona-Müller, Fernando Grover-Paez, Carlos Gerardo Ramos-Becerra, Ernesto Germán Cardona-Muñoz, Maria Guadalupe Ramos-Zavala, Jaime Carmona-Huerta, Jorge Eduardo Hernandez-del-Rio, Tomas Miranda-Aquino, Christian Gonzalez-Padilla, Christopher Josue Lopez-Gradilla

**Affiliations:** 1 Unidad de Cardiología, Del Hospital Civil Fray Antonio Alcalde, Guadalajara 44200, Mexicotomas_miranda_a@hotmail.com (T.M.-A.); 2 Instituto de Terapéutica Experimental y Clínica, Centro Universitario de Ciencias de La Salud, Universidad de Guadalajara, Guadalajara 44340, Mexico; fernando.grover@academicos.udg.mx (F.G.-P.); guadalupe.ramos@academicos.udg.mx (M.G.R.-Z.); jaime.camona@academicos.udg.mx (J.C.-H.); drjorgehdez@hotmail.com (J.E.H.-d.-R.); dr.christian.glez@gmail.com (C.G.-P.); christopher.lgradilla@alumnos.udg.mx (C.J.L.-G.)

**Keywords:** long COVID, cardiac arrhythmias, atrial fibrillation, Holter monitoring, metformin, post-acute sequelae of COVID-19

## Abstract

Background: Cardiac arrhythmias are a frequent complication of acute SARS-CoV-2 infection. However, their long-term prevalence and clinical determinants among patients with post-COVID-19 syndrome, especially those previously hospitalized, remain poorly defined. Objectives: To assess the prevalence and types of arrhythmias in long COVID patients following hospitalization and to identify associated clinical risk factors. Methods: In this cross-sectional study, 53 patients previously hospitalized with confirmed COVID-19 were evaluated ≥3 months post-infection. All participants underwent a standardized clinical assessment, 12-lead electrocardiography, and 24 h Holter monitoring. Logistic and Cox regression analyses were performed to identify predictors of arrhythmia. Results: Arrhythmias were identified in 41.5% (*n* = 22) of patients. Atrial fibrillation (32%) was the most frequent arrhythmia, followed by sinus bradycardia (27%) and sinus tachycardia (18%). Age (OR 1.06, 95% CI 1.01–1.10, *p* = 0.01) and length of hospital stay (OR 1.1, 95% CI 1.01–1.2, *p* = 0.04) were independently associated with arrhythmia. Biguanide (metformin) therapy was inversely associated with the occurrence of arrhythmia (Exp(B) = 0.017, *p* = 0.008). Dyspnea (82.4%) and palpitations (41.5%) were the most commonly reported symptoms. Conclusions: Arrhythmias are common in patients with long COVID following severe disease. Advanced age and prolonged hospitalization are significant risk factors, while biguanide use may offer a protective effect. These findings underscore the need for targeted cardiac surveillance in this population.

## 1. Introduction

Cardiac arrhythmias are defined as abnormalities in the rate or regularity of cardiac electrical activation, resulting in an irregular, excessively slow or fast heartbeat or abnormal propagation of electrical impulses through the myocardium [1]. Their causes and clinical consequences vary widely. Atrial arrhythmias are common in patients during SARS-CoV-2 infection and are associated with increased mortality and the need for mechanical ventilation [2]. Retrospective studies have shown that COVID-19 patients with atrial fibrillation or flutter have twice the mortality rate. Additionally, atrial fibrillation occurs in approximately 15% of hospitalized patients during their COVID-19 hospitalization [2].

Due to the SARS-CoV-2 pandemic, investigations are ongoing regarding the long-term cardiovascular manifestations and cardiovascular complications following a SARS-CoV-2 infection. The susceptibility, severity, and progression of cardiac arrhythmias resulting from SARS-CoV-2 are not yet fully understood. Therefore, it is crucial to determine the prevalence of cardiac arrhythmias in post-COVID-19 patients to take timely action.

Shortly after the first wave of the COVID-19 pandemic, it was observed that some patients presented persistent symptoms after the resolution of the acute infection. This has led to the use of different terms in the literature, such as prolonged COVID-19, acute post-COVID-19 syndrome, or post-COVID-19 disorders, without a standardized definition.

To standardize terminology, the WHO and the CDC propose the generic use of “conditions following COVID” as a broad umbrella for health consequences that persist beyond 4 weeks from the acute infection. The Center for Disease Control and Prevention (CDC) has formulated the term “post-COVID-19 conditions” to describe health issues that persist more than four weeks after being infected with COVID-19 [3]. These include the following:Long COVID (a wide range of symptoms that can last for weeks or months) or persistent post-COVID-19 syndrome (PPCS); Multiorgan effects of COVID-19; Effects of COVID-19 treatment/hospitalization.

On the other hand, the UK’s National Institute for Health and Care Excellence (NICE) defines the course of COVID-19 as an acute phase of up to 4 weeks (from symptom onset), an ongoing symptomatic phase from weeks 4–12, and post-COVID-19 syndrome from week 12 onward [4]. The definition of post-COVID-19 syndrome refers to symptoms and signs occurring during or after the acute COVID-19 infection that persist beyond 12 weeks from the onset of the infection and are not explained by an alternative diagnosis. Therefore, the term long COVID encompasses all manifestations attributable to COVID-19 that persist beyond the acute phase [3,4].

We carried out a cross-sectional study to determine the prevalence of arrhythmias as part of a long COVID manifestation in patients requiring in-hospital attention due to COVID-19 infection.

## 2. Material and Methods

Study Design: An observational, descriptive, cross-sectional study was conducted to determine the prevalence of and characterize cardiac arrhythmias during the post-acute phase following SARS-CoV-2 infection. Adult male and female patients aged 18–90 years with a documented history of COVID-19 at least three months before enrollment were eligible for inclusion. Previous infection was confirmed by review of institutional medical records, demonstrating a positive reverse transcription polymerase chain reaction (RT-PCR) or antigen test for SARS-CoV-2.

Study Population: Adults aged 18–90 years with laboratory-confirmed COVID-19 ≥ 3 months prior and previous hospitalization were consecutively recruited during routine cardiology follow-up visits. This includes residents of the metropolitan area of Guadalajara who were hospitalized due to COVID-19 and who voluntarily agreed to participate in the study and sign the informed consent form.

Study Site: The study was conducted at the Cardiology Department of the Hospital Civil de Guadalajara “Fray Antonio Alcalde,” in collaboration with the Instituto de Terapéutica Experimental y Clínica (INTEC) of the University of Guadalajara. Recruitment and clinical evaluations were performed within the cardiology outpatient clinic and diagnostic imaging units during the predefined study period. All assessments were performed by trained cardiology personnel following standardized institutional protocols.

Sample Size: The required sample size was calculated using a 95% confidence level and a 5% margin of error. The sample size calculation is based on determining the prevalence of arrhythmias in post-COVID-19 patients; Z corresponds to the value of statistical confidence, which is predetermined at 95%. For this confidence level, the Z-score is 1.96. The remaining parameters used varied according to the desired objective, considering previous literature reports. The prevalence of arrhythmia obtained from A. Dewland et al. [5] in post-COVID-19 patients, which was 4%, was used as a reference. The prevalence was *p* = 0.04 and ε was the 5% margin of error. The sample size was estimated using the formula *n* = Z^2^·P·(1 − P)/ε^2^, with *p* = 0.04, Z = 1.96, ε = 0.05, yielding a minimum of 59 patients with complete datasets.

### 2.1. Selection Criteria

Inclusion Criteria: Written informed consent form. Age 18–90 years. Laboratory-confirmed COVID-19 ≥ 3 months prior (positive antigen test or PCR).

Exclusion Criteria

- Physical or mental incapacity preventing participation in the study.
- Inability to undergo Holter monitoring. Pre-existing arrhythmia prior to SARS-CoV-2 infection. Withdrawal of informed consent during the testing period.
- Use of class I or III antiarrhythmics, beta-blockers, or non-dihydropyridine calcium channel blockers
- Known ischemic heart disease, dilated cardiomyopathy, or severe valvular disease.

### 2.2. General Study Description

Patients attended the cardiology department for clinical evaluation and testing. After signing informed consent, clinical data were recorded.

After verification of eligibility, participants underwent standardized clinical evaluation. Demographic and clinical data were collected using structured case report forms designed specifically for the study.

Recorded variables included age, sex, anthropometric measurements (weight, height, and body mass index), and cardiovascular symptoms including palpitations, dyspnea, chest pain, syncope, and presyncope. Documentation of previous SARS-CoV-2 infection was verified through review of institutional laboratory records. Cardiovascular Assessment: All participants underwent a standardized cardiovascular evaluation including resting electrocardiography, ambulatory rhythm monitoring, and transthoracic echocardiography. Electrocardiography: A standard resting 12-lead electrocardiogram was obtained using BeneHeart R3 Mindray equipment under standardized acquisition conditions. Recordings were analyzed by cardiology personnel to assess heart rhythm, heart rate, conduction abnormalities, PR, QRS, and QT intervals, and repolarization abnormalities. 24-Hour Ambulatory Holter Monitoring: Continuous ambulatory electrocardiographic monitoring was performed using a Contec Model TLC-9803 three-channel system for 24 h. Holter recordings were analyzed using dedicated software by trained cardiology staff. Detected arrhythmias were classified as supraventricular or ventricular according to established electrocardiographic criteria.

For the purposes of this study, arrhythmia was operationally defined as the presence of any of the following: Premature atrial contractions exceeding age-adjusted expected frequency; supraventricular tachycardia episodes; atrial fibrillation or flutter; premature ventricular complexes exceeding normal limits; ventricular tachycardia (sustained or non-sustained); sinus pauses greater than 2 s; second- or third-degree atrioventricular block. All Holter tracings with abnormal findings were independently reviewed and confirmed by a certified cardiologist to reduce misclassification bias. Transthoracic echocardiography was performed using a Siemens Acuson SC2000 system equipped with a P4-2 sector transducer, with patients in the left lateral decubitus position. Studies were conducted according to standardized echocardiographic guidelines. The evaluation included global and segmental left ventricular wall motion, left ventricular ejection fraction, diastolic function, morphological and functional assessment of cardiac valves, presence and severity of valvular disease, chamber dimensions and volumes, right ventricular systolic function, and estimated pulmonary artery systolic pressure.

### 2.3. Study Description

- Visit 1: Informed consent signing, medical history, clinical determinations, electrocardiogram, and placement of Holter monitor.
- Visit 2: Removal of the 24 h Holter monitor. An echocardiogram was performed.

### 2.4. Statistical Analysis

All collected data were entered into a structured electronic database created in Microsoft Excel for Mac Version 16.68. The dataset underwent systematic verification, double-checking, and validation procedures prior to statistical analysis. Holter and echocardiographic interpretations were reviewed by experienced cardiology personnel to ensure consistency. Only authorized members of the research team had access to the final dataset and to minimize selection bias, consecutive eligible patients attending cardiology follow-up were invited to participate. Standardized protocols were used for all diagnostic procedures to reduce measurement variability. Arrhythmia interpretation was confirmed by a certified cardiologist to decrease classification error. Exclusion of patients with pre-existing arrhythmia or structural heart disease reduced confounding related to baseline cardiovascular pathology.

### 2.5. Ethical Considerations

This project adheres to ethical standards, the General Health Law Regulations on Research, and the principles emanating from the 18th Medical Assembly of Helsinki, Finland in 1964 and subsequent modifications made by assemblies in Tokyo, Japan, in 1975; Venice, Italy, in 1983; Hong Kong in 1989, the 48th General Assembly of Somerset West, Republic of South Africa, in 1996; the 2nd General Assembly, Edinburgh, Scotland, October 2000; the clarification note added by the General Assembly of the World Medical Association, Washington 2002; the clarification note added by the WMA General Assembly, Tokyo 2004; the 59th General Assembly, Seoul, Korea, October 2008; and the 64th General Assembly, Fortaleza, Brazil, October 2013, which contemplate ethical principles for medical research in humans. The study was approved by the Ethics and Investigation Committee of the Hospital Civil de Guadalajara with the following registration: CEI18/23.

In accordance with good clinical practice guidelines, all study participants were identified only by initials and numbers in the electronic database. Data was stored confidentially, along with questionnaire responses and clinical and laboratory test results, to ensure privacy.

## 3. Results

**Figure 1:**
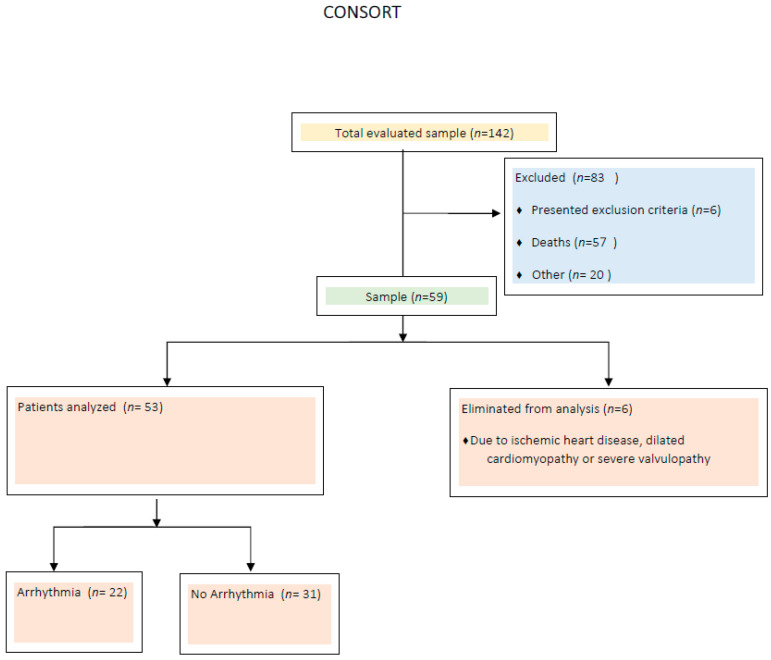
Study population selection and analysis.

A total of 142 patients that were hospitalized from January 2022 to February 2022 due to a SARS-CoV-2 diagnosis were identified through electronic records and census data from the infectious disease department. Of these, 57 patients did not survive hospitalization. The remaining patients were approached to participate in the study voluntarily, but 16 patients declined or were lost to follow-up, 6 patients met exclusion criteria, and 4 patients did not attend their scheduled appointments. Ultimately, 59 patients were recruited; 6 were excluded due to predefined criteria, leaving 53 patients for analysis (Figure 1). 

A total of 53 patients were analyzed, and their general characteristics were as follows: The average age was 58.13 ± 16.52 years, with the youngest patient being 19 years old and the oldest being 90 years old. Patients with arrhythmias were significantly older than those without (63.8 ± 16.1 vs. 52.6 ± 15.3 years). Women represented 54.7% of the sample.

For the analysis, the subjects were divided into two groups: those who had arrhythmia (*n* = 22), and those who did not have arrhythmia (*n* = 31). The general characteristics are summarized in Table 1 and the echocardiographic characteristics are shown in Table 2.

Within the patients’ history, 71.7% of the patients (38 patients) were taking medication for their comorbidities.

In Table 3 we show the distribution of the drugs that were used by the studied population

An arrhythmia was detected in 41% of the patients (*n* = 22). Thus, the prevalence of arrhythmias in this sample of previously hospitalized post-COVID-19 patients was 41%. Among patients with arrhythmias, 45% (*n* = 10) presented with bradyarrhythmia and 55% (*n* = 12) with tachyarrhythmias. (Table 4).

The percentage of each was classified according to their general classification (bradyarrhythmia or tachyarrhythmia, (Table 4). It is worth mentioning that one patient had both atrial tachycardia and atrial fibrillation with slow ventricular response. Another patient had atrial fibrillation with slow ventricular response, sinus bradycardia, and complete atrioventricular block.

Atrial fibrillation was the most prevalent arrhythmia, found in seven of the patients representing 32% of the total arrhythmia presented. One patient had both atrial tachycardia and slow ventricular response atrial fibrillation. Another patient had slow ventricular response atrial fibrillation, sinus bradycardia, and third-degree atrioventricular block.

To determine the association between different variables and the development of arrhythmias, univariate and multivariate logistic regression analyses were conducted. Variables with a *p*-value less than or equal to 0.2 were included in the multivariate analysis. A statistical significance level of *p* < 0.05 was considered. Risk association findings for developing arrhythmias included age with a significant *p*-value of 0.01 and length of hospital stay with a *p*-value of 0.04 (Table 5).

Furthermore, we analyzed the association of the different analyzed variables as a risk factor for the development of arrhythmias with a Cox regression, which is shown in Table 6.

## 4. Discussion

Since the beginning of the pandemic, cardiovascular disorders have been among the most common extrapulmonary manifestations of SARS-CoV-2 infection [6]. Approximately 20–30% of hospitalized COVID-19 patients develop cardiac complications, which are associated with increased mortality. Cardiac involvement in acute COVID-19 has a variety of patterns, ranging from asymptomatic elevations in cardiac biomarkers to cardiogenic shock and sudden cardiac death [7]. There is now increasing evidence that these disorders may provide a basis for subsequent cardiac arrhythmias among patients who have experienced a SARS-CoV-2 infection [8]. Our study found a 41% prevalence of arrhythmias among previously hospitalized post-COVID-19 patients. This contrasts with Dewland et al., who reported a low incidence of clinically significant arrhythmias, with no presence of atrial fibrillation, atrial flutter, supraventricular tachycardia, or third-degree atrioventricular block. They found a 20% presence of sinus tachycardia. However, in this study, only 8% of the patients were hospitalized due to COVID-19, and the vast majority were clinically classified as mild cases. A third of the patients presented palpitations, which is similar to our results with 41%.

On the other hand, the study conducted by Aponte et al. [9], which assessed the presence of ventricular arrhythmia in patients with post-COVID-19 syndrome, found that 31.65% of patients had mild disease, 59.5% had moderate disease, and 8.86% had severe disease. The most frequent types of arrhythmias according to the type of COVID-19 suffered were: sinus rhythm (28%) in mild cases, sinus tachycardia (26%) for moderate cases, and ventricular tachycardia (43%) in severe cases. Inappropriately fast sinus tachycardia was the most prevalent arrhythmia, followed by atrial fibrillation. In our study, atrial fibrillation was the most prevalent cardiac arrhythmia, which differs from the latter report. In a retrospective study, Al-Aly Z. et al. [10] also identified an increase in the prevalence of arrhythmias in the first 6 months after COVID-19 infection. They evidenced an increase of 1.7 times in the incidence of atrial fibrillation in the Veterans Health Administration electronic medical record. Consistent with previous trials, these data demonstrate the reproducibility of our results.

Regarding the findings from the Holter monitor and electrocardiogram in post-COVID-19 patients, a higher percentage of tachyarrhythmia (55%) was found. It is known that during the acute phase, the SARS-CoV-2 virus infection has the potential to trigger cardiac rhythm disorders in patients without a history of arrhythmia or structural heart disease as an arrhythmogenic substrate. Multiple inflammatory cytokines with increased expression during the acute stage of COVID-19 infection, like interleukin-6 (IL-6) and tumor necrosis factor-α, can directly modify the expression and function of cardiac potassium and calcium channels, affecting the myocyte action potential [11]. Also, the induction of direct cardiac damage, myocarditis, myocardial ischemia, and heart failure are possible causes for the development of arrhythmias [7,8]. Chronic arrhythmias associated with COVID-19 are less well understood. Growth factors such as TGF-β and PDGF, along with persistent inflammatory cytokine exposure (TNF-α, IL-1, IL-6, IL-10, and IL-4) may promote myocardial fibrosis and remodeling, creating an arrhythmogenic substrate [12,13]. The presence of chronic active myocarditis, in which recovery from the initial inflammatory phase does not occur and disease activity persists due to viral genome persistence and possible immune dysregulation, may also explain the persistence of arrhythmias in this patient population, as this sustained inflammatory state maintains an arrhythmogenic substrate. In addition, patients who have experienced viral myocarditis may develop post-inflammatory myocardial scarring, which could further explain the persistence of arrhythmias in a chronic setting [14]. In a small study, Puntmann V.O et al. [15] identified image alterations in 58% of post-COVID-19 recovered patients. The cardiac magnetic resonance revealed myocardial edema and scarring on late gadolinium enhancement. These findings at least explain some of the possible pathophysiological alterations found after COVID-19 infection that can partially explain the high prevalence of arrhythmia in this group of patients.

In addition to demonstrating the prevalence of arrhythmias in hospitalized post-COVID-19 patients, the aim was to determine the association of different clinical variables with the development of arrhythmias. Age (65 ± 17) was found to have a statistically significant association for the development of arrhythmias, which had not been previously reported in similar studies but had for presenting severe COVID-19. The length of hospital stay was also statistically associated with arrhythmia development (11 ± 9), suggesting a direct proportional association between the length of hospital stay and arrhythmia development. In the study by Wang and colleagues [16], cardiac arrhythmias related to COVID-19 were described for the first time, reporting an incidence of arrhythmias of 17% (23/138), 16 of whom were admitted to the intensive care unit (ICU), representing 44% of the total number of patients in the ICU. These patients also had longer lengths of hospital stay.

In our study, diabetes mellitus appeared to be associated with a lower risk of arrhythmias (*p* = 0.01). However, it is known that diabetes mellitus increases the risk of coronary artery disease and mortality compared to the general population [17]. Recently, a publication by Sunyoung Kim et al. [18] partially addressed this issue. They analyzed a total of 2,515,268 patients with type 2 diabetes and determined the association between the treatment of diabetes and the incidence of arrhythmias. They concluded that the use of metformin and thiazolidinediones are effective for preventing AF in patients with type 2 diabetes, making it plausible that this protective effect is likely attributable not to diabetes, but to the antidiabetic therapy, particularly metformin. Also, other authors like Lee TTL et al. [19] showed an increased risk of ventricular arrythmias and death in patients with type 2 diabetes that were treated with sulfonyl urea compared to metformin. Our Cox regression analysis confirmed a protective association between biguanide use and arrhythmia occurrence.

Part of this antiarrhythmic effect can be attributed to the different molecular mechanisms triggered by metformin. Metformin exerts antiarrhythmic effects through several interrelated molecular mechanisms, primarily mediated by the activation of AMP-activated protein kinase (AMPK) [20]. This activation leads to improvements in calcium homeostasis, attenuation of oxidative stress and inflammation, and prevention of adverse atrial structural and electrical remodeling. Specifically, metformin increases the expression of connexin-43 (Cx43), a key gap junction protein, which enhances electrical conduction in the atria [21]. It also restores the function of small conductance calcium-activated potassium (SK) channels by normalizing the expression of SK2 and reducing SK3, thereby stabilizing the action potential duration [22]. In models of rapid atrial pacing and diabetes, metformin reduces atrial fibrosis and lipid accumulation via pathways involving PPARα and VLCAD and suppresses pro-inflammatory cytokines such as TNF-α and TGF-β1 [23]. These actions collectively reduce atrial effective refractory period (AERP) dispersion and prevent the perpetuation of atrial fibrillation. In the context of ventricular arrhythmias, metformin improves myocardial energy metabolism, preserves ATP levels, reduces QT dispersion, and increases phosphorylated Cx43 expression, particularly when combined with agents like vildagliptin [24]. These mechanisms demonstrate metformin’s multifaceted role in reducing arrhythmogenic substrates and stabilizing cardiac electrophysiology.

The pathophysiology of atrial fibrillation (AF) is increasingly recognized as an inflammatory process. Elevated levels of high-sensitivity C-reactive protein (hs-CRP) are consistently correlated not only with the incidence and persistence of AF but also with a heightened risk of recurrence following cardioversion or ablation [25]. This inflammatory link is particularly relevant in the context of COVID-19, where elevated hs-CRP has been identified as a robust predictor of adverse clinical outcomes and increased mortality [26]. Consequently, COVID-19—much like other systemic inflammatory conditions—significantly escalates the risk of developing AF. Therapeutic interventions aimed at mitigating this inflammatory surge, such as metformin, pioglitazone, linagliptin, statins, and colchicine, have shown potential in reducing hs-CRP levels and, by extension, the incidence of AF [21,27].

Other comorbidities such as hypertension, obesity, hypothyroidism, and chronic kidney disease were not associated with the development of arrhythmias in our studied population.

Among the cardiovascular symptoms, shortness of breath was the most prevalent at 79%, followed by palpitations at 49%, which aligns closely with the findings by Aponte [8] and Davis [28] with their studies revealing higher percentages of the same symptoms.

## 5. Study Limitations

This cross-sectional study aimed to determine the prevalence of arrhythmias as a cardiovascular manifestation of long COVID. Due to the cross-sectional design, causal relationships cannot be established. Furthermore, while we analyzed key variables potentially associated with arrhythmogenesis, unmeasured confounders may have influenced the findings. One of these key limitations includes the absence of prior Holter monitoring; therefore, the presence of previously existing asymptomatic arrhythmias cannot be ruled out, and the prevalence of rhythm disturbances in this patient group may be overestimated. Consequently, further research is needed to confirm or refute these results.

For future research directions, a larger sample could be taken to confirm these findings as well as a prospective study to determine the causality of severe COVID-19 and arrhythmias. It would be interesting to identify the potential antiarrhythmic effect of metformin in a prospective trial, not only in patients that suffered from COVID-19 but also in patients at an elevated risk of developing arrhythmia.

## 6. Conclusions

Among patients previously hospitalized for severe COVID-19, the prevalence of arrhythmias during long COVID was 41%. Age and duration of hospitalization were associated with arrhythmia risk. Metformin use may exert a protective effect in this group of individuals.

## Figures and Tables

**Table 1:** General characteristics.

Variable	Global	Arrhythmia	Controls	*p* *
n	53	22(41%)	31(59%)	
Age	58 (±16)	65 (±17)	53 (±14)	0.006
Gender (female)	29 (55%)	14 (63%)	15 (48%)	0.1
Type 2 diabetes	20 (38%)	5 (22%)	15 (48%)	0.1
Arterial hypertension	26 (49%)	9 (41%)	17 (55%)	0.5
Smoker	19 (36%)	8 (36%)	11 (35%)	0.8
Obesity	23 (43%)	10 (45%)	13 (42%)	0.9
CKD	4 (8%)	1 (5%)	3 (10%)	0.9
COPD	4 (8%)	3 (14%)	1 (3%)	0.4
Hypothyroidism	2 (4%)	0 (0%)	2 (6%)	0.6
Length of hospital stay	8 (±6)	11 (±9)	6 (±4)	0.04
ICU	2 (4%)	1 (5%)	1 (3%)	0.6
Invasive mechanical ventilation	2 (4%)	1 (5%)	1(3%)	0.6
High oxygen flow	12 (23%)	7(32%)	6 (16%)	0.3
O_2_ (nasal cannulae)	39 (74%)	14 (63%)	25 (80%)	0.3
HIV	1 (1.9%)	1 (3.8%)	0 (0%)	0.491
SBP	126.0 ± 21.0	123.9 ± 21.3	128.0 ± 20.9	0.489
DBP	73.7 ± 11.2	74.4 ± 10.5	73.0 ± 12.0	0.648
Heart rate	74.5 ± 16.9	74.3 ± 22.9	74.6 ± 8.4	0.959
BMI	28.8 ± 6.2	27.8 ± 7.0	29.8 ± 5.2	0.252
Overweight	14 (26.4%)	5 (19.2%)	9 (33.3%)	0.352
Obesity	22 (41.5%)	10 (38.5%)	12 (44.4%)	0.782
Palpitations	22 (41.5%)	16 (61.5%)	6 (22.2%)	0.005
Dyspnea	42 (82.4%)	22 (91.7%)	20 (74.1%)	0.100
Chest pain	8 (15.7%)	5 (20.8%)	3 (11.1%)	0.451
Syncope	5 (9.8%)	5 (20.8%)	0 (0%)	0.018
Fainting	8 (15.7%)	6 (25.0%)	2 (7.4%)	0.127

CKD: chronic kidney disease, COPD: chronic obstructive pulmonary disease, ICU: intensive care unit, SBP: systolic blood pressure, DBP: diastolic blood pressure, BMI: body mass index. * Mann–Whitney U test.

**Table 2:** Echocardiographic characteristics.

Characteristics	All	Arrhythmia	Control (No Arrhythmia)	*p*
n	53	22(41%)	31(59%)	
Left ventricular ejected fraction	60.5 ± 6.5	60.7 ± 4.7	60.4 ± 7.9	0.854
Strain LV, %	−19.3 ± 2.6	−19.2 ± 2.2	−19.3 ± 3.0	0.945
Strain LVEF, %	57.7 ± 6.7	58.9 ± 5.5	56.8 ± 7.5	0.283
PASP, mmHg	32.3 ± 11.5	33.1 ± 14.4	31.6 ± 8.1	0.630
Cardiac reservoir,	37.7 ± 13.8	38.9 ± 11.9	36.5 ± 15.6	0.534
Pump	17.2 ± 6.2	16.9 ± 4.1	17.6 ± 7.7	0.704
Conduct	20.5 ± 10.3	22.1 ± 10.0	18.9 ± 10.5	0.265

LV: left ventricle, LVEF: left ventricular ejection fraction, PASP: pulmonary artery systolic pressure.

**Table 3:** Drug use profile.

	All	Arrhythmia	Control (No-Arrhythmia)	*p*
Drugs	38 (71.7%)	18 (69.2%)	20 (74.1%)	0.766
Angiotensin-converting enzyme inhibitors	8 (15.1%)	5 (19.2%)	3 (11.1%)	0.467
Angiotensin II receptor antagonists	11 (20.8%)	3 (11.5%)	8 (29.6%)	0.175
Thiazide	3 (5.7%)	1 (3.8%)	2 (7.4%)	>0.999
CCB (DHP)	6 (11.3%)	1 (3.8%)	5 (18.5%)	0.192
Sulfonylurea	3 (5.7%)	0 (0%)	3 (11.1%)	0.236
Biguanide	16 (30.2%)	3 (11.5%)	13 (48.1%)	0.006
Insulin	4 (7.5%)	2 (7.7%)	2 (7.4%)	>0.999
Antiepileptic	1 (1.9%)	0 (0%)	1 (3.7%)	>0.999
Lipid-lowering	0 (0%)	0 (0%)	0 (0%)	-----

CCB: calcium channel blockers, DHP: dihydropyridines.

**Table 4:** Arrhythmia distribution.

Arrhythmia	Subjects (*n* = 53)
Bradyarrhythmia	10 (45%)
Sinus bradycardia	6 (60%)
Third-degree atrioventricular block	4 (40%)
Slow atrial fibrillation	2 (20%)
Tachyarrhythmia	12 (55%)
Sinus tachycardia	4(33%)
Atrial fibrillation	5 (42%)
Supraventricular tachycardia	1 (8%)
Atrial flutter	2 (16%)
Atrial tachycardia	1 (8%)

**Table 5:** Univariate and multivariate analysis for arrhythmia development.

Variable	Univariate OR (CI95%)	*p*	Multivariate OR (CI95%)	*p*
Age	1.05 (1.01–1.1)	0.001	1.06 (1.01–1.1)	0.01
Sex (female)	1.8 (0.6–5.7)	0.3		
Diabetes mellitus	0.3 (0.1–1.1)	0.06	0.2 (0.1–0.7)	0.01
Systemic hypertension	0.6 (0.2–1.7)	0.3		
Tobacco	1.03 (0.3–3.2)	0.9		
Obesity	1.2 (0.4–3.5)	0.8		
Chronic kidney disease	0.4 (0.1–4.5)	0.5		
COPD	4.8 (0.5–48)	0.2	1.4 (0.2–41)	0.5
Length of hospital stay	1.08 (0.99–1.2)	0.07	1.1 (1.01–1.2)	0.04
ICU	1.4 (0.1–24)	0.8		
Invasive mechanical ventilation	1.4 (0.1–24)	0.8		
High oxygen flow	2.4 (0.6–9.1)	0.2	2.5 (0.2–29)	0.8
O_2_ with mask	0.4 (0.1–1.4)	0.2	0.8 (0.1–35)	0.7

COPD: chronic obstructive pulmonary disease, ICU: intensive care unit.

**Table 6:** Forward log regression.

	*p*	Exp(B)	95% C.I. for EXP(B) Low–High
Age	0.023	5.833	1.008–1.108
Palpitations	0.014	6.980	1.477–33.000
Biguanide	0.008	−2.334	0.017–0.543
Constant	0.019	NA	NA

## Data Availability

The data can be made available by requesting it to david.cardona@academicos.udg.mx.
